# Hand-Book of Practical Medicine

**Published:** 1886-08

**Authors:** 


**Hand-book of Practical Medicine.** By DR. HERMANN EICHHORST, Professor of Special Pathology and Therapeutics, University Medical Clinic, in Zurich; 103 wood engravings. New York: Wm. Wood & Co., 56 and 58 Lafayette Place. 1886.

The above belong to "Wood's Library," and they are valuable additions to the admirable volumes which have appeared in the series of this year. The works of the great Hippocrates have been unobtainable by the majority of the profession, and this excellent translation will be read with no little interest. Those who care only for the practical side of medicine, will be well satisfied with Prof. Eichhorst's hand-book. This first volume is devoted to diseases of the circulatory and respiratory apparatus. The plan of the work is an admirable one, well calculated to meet the wants of the busy practitioner. We look forward to the appearance of the second volume with much interest.

